# Physical function and quality of well-being in fibromyalgia: the applicability of the goodness-of-fit hypothesis

**DOI:** 10.1080/21642850.2014.905205

**Published:** 2014-04-28

**Authors:** Maya S. Santoro, Charles Van Liew, Terry A. Cronan, Heather M. Franks, Rebecca N. Adams, Scott C. Roesch, Jennalee S. Wooldridge, Mitsuo Tomita

**Affiliations:** ^a^ San Diego State University/University of California, San Diego Joint Doctoral Program in Clinical Psychology, 5500 Campanile Drive, San Diego, CA, USA, 92120; ^b^ Department of Psychology, San Diego State University, San Diego, CA, USA; ^c^ Kaiser Permanente of Southern California, San Diego, CA, USA

**Keywords:** fibromyalgia syndrome, quality of well-being, physical function, coping, control

## Abstract

*Objective*: The goodness-of-fit hypothesis suggests that the effectiveness of a coping strategy depends on the match between type of strategy (problem-focused, emotion-focused) and the level of perceived control. This hypothesis was examined as a predictor of physical functioning and quality of well-being (QWB) in a large sample of women with fibromyalgia. *Methods:* Participants were 478 women with diagnosed fibromyalgia (*M*_age_ = 54.31, SD = 11.2), who were part of a larger intervention in which no intervention effects were found. Hierarchical, mixed selection regressions were performed to determine whether the relationship between coping and control-predicted physical functioning and QWB. *Results:* Participants who reported having lower levels of perceived control over their fibromyalgia syndrome and who engaged in more self-controlling coping (emotion-focused strategy) experienced greater QWB and physical functioning than those who used less self-controlling coping. Various main effects for coping and perceived control were also found. Level of physical functioning was also related to escape-avoidance, distancing, and perceived control. The level of QWB was related to social-support seeking, accepting responsibility, distancing, problem-solving, and perceived control. *Conclusions:* This study provides a greater understanding of the relationships among coping, perceived control, physical functioning, and well-being for women with fibromyalgia. Implications and directions for future research are discussed.

## Introduction

Fibromyalgia syndrome (FMS) is a chronic, painful condition that affects approximately 2% of the population (Lawrence et al., 2008; Wolfe, Hauser, Hassett, Katz, & Walitt, 2011). It is characterized by widespread musculoskeletal pain, stiffness, disrupted sleep, and fatigue. There is no agreed-upon etiology or biological marker for FMS. Given that FMS has no cure, and that pain and problems in physical functioning are not eliminated with medication, it is likely that many people believe that they have little control over their FMS. Lazarus and Folkman (1984) have suggested that the effectiveness of a coping strategy is related to one's perception of control over a stressful event. The goodness-of-fit hypothesis suggests that a coping strategy is most effective when it matches the level of perceived control one believes he or she has. In the present study, the goodness-of-fit hypothesis was examined for individuals with FMS. People with FMS serve as an appropriate test for the goodness-of-fit hypothesis because FMS is particularly burdensome and is often associated with feelings of helplessness or lack of control over the effects of the syndrome (Martinez, Ferraz, Sato, & Edgard, 1995). In addition, feeling a sense of control over the FMS may have an important influence on quality of life (Boyer et al., 2010).

### Coping

Coping can be described as a cognitive and/or behavioral attempt to manage situations and internal states that are stressful to the individual (Folkman & Moskowitz, 2005). Folkman and Lazarus (1984) defined two types of coping strategies: problem-focused and emotion-focused. Problem-focused coping was defined as methods for managing external demands and conflicts, and emotion-focused coping was defined as methods for managing internal demands and unpleasant emotions that emerge in stressful situations.

Previous research on coping with chronic illness has suggested that problem-focused coping is more often related to positive health outcomes (Felton, Revenson, & Hinrichsen, 1984), and emotion-focused coping is more often associated with poorer health outcomes (Bombardier, D'Amico, & Jordan, 1990). However, several studies have suggested that additional factors may affect the relationship between coping and health outcomes, e.g. the amount of perceived and actual control one has (Folkman & Moskowitz, 2005; Stanton, Kirk, & Cameron, 2000).

### Control

Perceived control can be described as the belief that one can alter his or her environment, behavior, internal states, and/or outcomes (Folkman, 1984). High levels of perceived control are often associated with positive outcomes, and high levels of helplessness are often associated with negative outcomes (Stiegelis et al., 2003). A sense of control is important for people with chronic illnesses because people who believe that they have control over their illness may put forth more effort toward problem-solving (Ross & Mirowsky, 1989) and experience less physiological response to stress (Brosschot et al., 1998).

### The goodness-of-fit hypothesis

The goodness-of-fit hypothesis, introduced by Folkman, Schaefer, and Lazarus (1979), predicts that coping produces better psychological adjustment when there is a fit between perceived controllability of an event and the type of coping strategy used to manage it. When a situation is perceived as controllable, problem-focused coping is thought to produce better outcomes. When a situation is appraised as uncontrollable, emotion-focused coping is regarded as the more effective coping strategy. The goodness-of-fit hypothesis might be most applicable when a person's coping resources are severely taxed (Park, Folkman, & Bolstrom, 2001), and people with FMS may be severely taxed because of the unpredictable and largely uncontrollable nature of their condition.

### Quality of well-being and physical function

Health-related quality of well-being (QWB) is primarily affected by health symptoms and functional status (Kaplan & Anderson, 1990). These factors promote or limit a person's ability to engage in tasks or activities that are necessary or enjoyable. Chronic pain is one symptom that is intimately related to activity limitation and poor physical and psychological well-being (Gureje, Von Korff, Simon, & Gater, 1996). Pain, in addition to the myriad of other FMS symptoms, negatively affects one's QWB (Bennett, 2005). In fact, findings suggest that functional QWB is very poor among individuals with FMS (Kaplan, Schmidt, & Cronan, 2000), and QWB is lower for those with FMS than for the general population (Campos & Vazquez, 2012), or for individuals with other serious chronic conditions (Kaplan et al., 1989).

The present study examined the applicability of the goodness-of-fit hypothesis in a sample of women with FMS to determine whether the fit between perceived control and coping strategy was related to their QWB and physical function.

## Materials and methods

### Participants

Participants were 478 women diagnosed with FMS (*M*_age_ = 54.31, SD = 11.2), who were part of a larger intervention in which no differences between intervention groups were found. Informed consent was obtained from all participants in the original study. Data from the six-month assessment period were used in the present study because coping was not assessed at the baseline assessment. Participants were primarily Caucasian (86%), married (64.4%), and employed (47.3%), and nearly 82% had attended at least some college.

### Measures

#### Coping

Coping was assessed via the revised Ways of Coping (WOC) questionnaire (Folkman & Lazarus, 1988), which is a self-administered survey consisting of 66 items, grouped into eight subscales to identify problem-focused and emotion-focused coping strategies. The problem-focused coping scale comprises the following sub-scales: confrontive coping, seeking social support, accepting responsibility, and painful problem-solving. The emotion-focused coping scale comprises the following sub-scales: distancing, self-controlling, escape-avoidance, and positive reappraisal. The internal consistency of the WOC, using standardized items, was 0.93 for the whole scale. Subscales yielded 0.59 ≤ *α* ≤ 0.79 within the present sample (confrontive coping = 0.61, distancing = 0.77, self-controlling = 0.59, seeking social support = 0.74, accepting responsibility = 0.60, escape-avoidance = 0.75, problem-solving = 0.74, positive reappraisal = 0.79). Construct validity for this measure has been supported by consistency with theoretical predictions (White, Richter, & Fry, 1992).

#### Control

The Arthritis Helplessness Index (AHI; Nicassio, Wallston, Callahan, Herbert, & Pincus, 1985) was used to assess control. This scale has often been used as a measure of perceived control (Brady, 2003; Nicassio, Kay, Custodio, Irwin, & Weisman, 2011). The scale was adapted for the FMS population by changing the term “arthritis” to “fibromyalgia”. The AHI is a self-administered measure of participants' perceptions of helplessness in coping with arthritis, or in this case, with FMS. The AHI assesses the belief that patients’ own behavior can control their disease (e.g. “If I do all the right things, I can successfully manage my fibromyalgia”, and “I can reduce my pain by staying calm and relaxed”), and assesses the level of control the patient feels he/she has, which is related to disease outcome expectancies (e.g. “Fibromyalgia is controlling my life” and “No matter what I do or how hard I try, I just can't seem to get relief from my pain”.). The questionnaire has a test–retest reliability of 0.53 over a 12-month period (Nicassio et al., 1985). The scale's internal consistency was 0.41 within the present sample if the scaling was taken as is. With reversals applied to allow for the fact that the AHI has been found to consist of two negatively related subscales (Stein, Wallston, & Nicassio, 1988), internal consistency was *α* = 0.82.

#### Quality of well-being

QWB was measured using the quality of well being (QWB) Scale. This scale was designed to measure general functioning. It includes four weighted subscales of function: symptom complex, mobility, physical activity, and social activity, and assesses the presence of 27 symptoms or problems over the past 6 days. A total QWB score was used for analyses in the present study. Reliability for the QWB has been demonstrated (Anderson, Kaplan, Berry, Bush, & Rumbaut, 1989), and validity has been shown for various conditions (Kaplan et al., 1989), including FMS (Kaplan et al., 2000). The scale was shown to have excellent internal consistency within the present sample (*α* = 0.98).

#### FMS impact on physical function

The Fibromyalgia Impact Questionnaire (FIQ; Burckhardt, Clark, & Bennett, 1991) is a self-administered questionnaire comprising 10 subscales that assess disease impact on physical functioning and psychological, social, and global well-being in people with FMS. This study examined only the physical function subscale, which comprises 10 items that measure functioning in everyday tasks during the past week, such as preparing meals and doing laundry. This subscale has been used on its own across studies as a measure of functionality or physical impairment (Bennett, 2005). The FIQ is reliable (*r* values ranged from 0.56 for pain to 0.95 for function) and valid for people with FMS (Burckhardt et al., 1991). The physical function subscale was shown to have excellent internal consistency within the present sample (*α* = 0.91). For scale-level descriptives for all measures, see Table 1.

**Table 1.** Scale level descriptive statistics (Non-standardized scores).

Scale	Mean	SD	Min	Max
WOC confrontive coping	0.8396	0.4447	0	2.6667
WOC seeking social support	1.0204	0.5983	0	2.8333
WOC accepting responsibility	1.4635	0.4545	0.2857	2.8571
WOC painful problem-solving	1.4338	0.5737	0	3
WOC distancing	1.0392	0.5729	0	3
WOC self-controlling	0.7798	0.5302	0	2.5
WOC escape/avoidance	1.5300	0.5855	0	3
WOC positive reappraisal	1.4112	0.6498	0	3
AHI^a^	1.4679	0.7248	−1.0909	3.4545
FIQ physical functioning	1.3408	0.7108	0	2.9
QWB	0.5619	0.0763	0.397	0.930

Note: WOC, Ways of coping questionnaire; AHI, Arthritis Helplessness Index; FIQ, Fibromyalgia Impact Questionnaire; QWB, quality of well-being scale.

^a^Reversals allowed for AHI items with negative signs for the scale.

### Procedure

The participants were from a larger study that measured the effects of social support and education on health care use and QWB in people with FMS (Oliver, Cronan, Walen, & Tomita, 2001). Participants were recruited through newspaper advertisements, mass mailings to members of a Health Maintenance Organization, fliers posted in physicians' offices, and physician referrals. To be eligible, participants had to the meet the American College of Rheumatology diagnostic criteria for FMS (Wolfe et al., 1990). Participants completed a series of questionnaires at baseline, 6 months, 1 year, and 18 months following the initial recruitment. The data for the present study were from the six-month assessment, because it was the only assessment that included a measure of coping.

#### Data analyses

To test whether the goodness-of-fit hypothesis would be supported, hierarchical, mixed selection regressions were performed using Stata 12.1 within four different levels: (1) a baseline, demographics model, (2) a first-level, main effects model, (3) a second-level, quadratic effects model, and (4) a third-level, interaction effects model. In the baseline model, age (mean centered), ethnicity (White or non-White; contrast coded 0.5, −0.5, respectively), employment status (gainfully employed or not gainfully employed; contrast coded 0.5, −0.5, respectively), family income (incremental, ranged values coded from 0 to 7 to abet interpretability), and time since diagnosis (in years and months) were entered to determine whether any of these variables were significant predictors of physical functioning (FIQ subscale) or QWB. All assessment-based predictors were standardized to assist in interpretability and avoid collinearity problems. In the first-level model, the main effect terms for the problem-focused and emotion-focused coping strategies (eight in total) and the main effect term for the control variable were considered. In the second-level model, quadratic terms for each coping strategy and the control variable were considered. In the third-level model, interaction effects were tested for each coping strategy (and the significant quadratic terms from the second-level models) by the control variable.

## Results

### Model selection

Given that the final models for each level were not nested fully because of the mixed method of selection, Akaike's and Bayes's Information Criteria (AIC and BIC) and adjusted *R* ^2^ were evaluated to determine which of these models yielded best fit of the data (Table 2; James, Witten, Hastie, & Tibshirani, 2013). For the FIQ physical functioning analyses, all criteria selected the Level 3 model. For the QWB analyses, AIC and adjusted *R* ^2^ selected the Level 3 model and BIC selected the Level 1 model. The model with the majority of information criteria selection was chosen for interpretation. Thus, the Level 3 model was interpreted for both outcomes.

**Table 2.** Information criteria for models.

	FIQ			QWB		
	AIC	BIC	Adj. *R*^2^	AIC	BIC	Adj. *R*^2^
Baseline	977.1624	997.9684	0.0995	5403.775	5424.581	0.1140
Level 1	858.8461	888.004	0.3040	5335.108	5368.431^a^	0.2704
Level 2	846.0383	887.6925	0.3267	5330.529	5384.679	0.2848
Level 3	841.7823^a^	887.6019^a^	0.3341^a^	5325.069^a^	5375.054	0.2915^a^

Note: FIQ,  Fibromyalgia Impact Questionnaire physical subscale; QWB, quality of well-being scale; AIC, Akaike's Information Criterion; BIC, Bayes's Information Criterion.

^a^Model judged best by respective criterion.

#### Third-Level model: significant demographics, main effects, and quadratic effects plus interaction effects

For the interaction models, all coping terms and control were re-introduced to permit testing the polynomial functions for each of them; however, only the significant quadratic terms from the second-level models were included. From the second-level model, the quadratic relationships between *functioning* and distancing and *functioning* and self-control were significant. For distancing, there was a significant linear trend, *β* = −0.2305, *t* = −6.60, *p* < .001, 95% CI [−0.2991, −0.1618], *sr* ^2^ = 0.0617, that demonstrated that as distancing increased, physical functioning improved. However, the quadratic effect of distancing, *β* = 0.0643, *t* = 2.57, *p* = 0.01, 95% CI [0.0152, 0.1134], *sr* ^2^ = 0.0094, demonstrated that, although physical functioning improved as distancing increased in general, at the highest levels of distancing, physical functioning began to decline again. There was not a significant linear trend of self-control strategies, *β* = −0.0006, *t* = −0.02, *p* = 0.984; however, there was a significant quadratic effect, *β* = 0.0518, *t* = 2.77, *p* = 0.006, 95% CI [0.0150, 0.0886], *sr* ^2^ = 0.0109. Visual analysis demonstrated that the best-fit quadratic line between self-control strategies and physical functioning was parabolic. At the lowest levels of self-control, physical functioning was poor; at moderately high levels of self-control (i.e. approximately 0.3 SD above mean), physical functioning was improved; and, at the highest levels of self-control, physical functioning was poor again.

With respect to QWB at the second level, the linear relationships between problem-solving, *β* = −2.1957, *t* = −0.50, *p* = 0.620, self-control, *β* = 6.6943, *t* = 1.58, *p* = 0.114, and reappraising, *β* = −1.3553, *t* = −0.29, *p* = .771, were non-significant. However, each of these was related to QWB significantly in quadratic form: problem-solving, *β* = 8.2261, *t* = 3.05, *p* = .002, 95% CI [2.9225, 13.5297], *sr* ^2^ = 0.0140, self-control, *β* = −5.9864, *t* = −2.54, *p* = .012, 95% CI [−10.6249, −1.3479], *sr* ^2^ = 0.0097, and reappraising, *β* = −6.3535, *t* = −2.18, *p* = .03, 95% CI [−12.0819, −0.6251], *sr* ^2^ = 0.0072. For problem-solving, the best-fit quadratic trend demonstrated that low levels were associated with high QWB scores, moderate (i.e. approximately mean) levels were associated with poorest QWB scores, and highest levels were associated with highest QWB scores. The best-fit quadratic line for self-control showed that low levels of self-control were related to low QWB, moderate (i.e. approximately mean) levels were related to highest QWB, and high levels of self-control were related to lowest QWB. For reappraising, lowest QWB was predicted by lowest levels, highest QWB was predicted by moderately high values (i.e. approximately .5 *SD* above the mean), and lower QWB was predicted by high levels of reappraisal.

**Figure 1.**
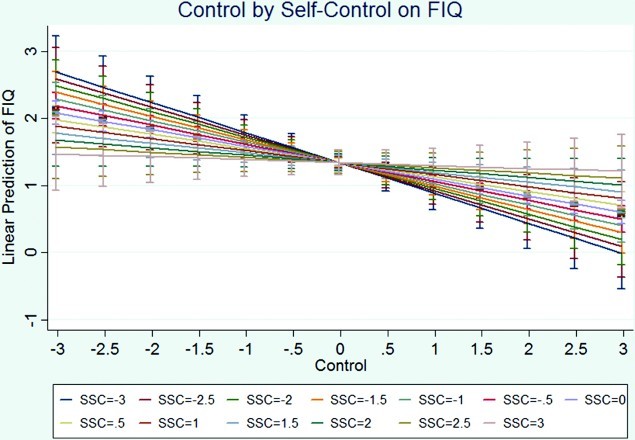
Visual representation of the interaction effect of self-control and control on physical functioning.

Note: FIQ,  Fibromyalgia Impact Questionnaire physical functioning subscale; SSC, standardized self-control.

For the third-level model, physical functioning was predicted by income, employment, age, distancing, self-control, escape-avoidance, and control, *F*(10,465) = 24.83, *p* < .0001, *R* ^2^ = 0.3481, RMSE = 0.5792. After controlling for the other variables in the model, the results indicated that those who had higher income, who were employed, who were older, who used escape-avoidance strategies less, and who felt more control experienced greater functioning (i.e. less FMS interference in functioning). The linear trend and the quadratic effect of distancing remained the same substantively. The non-significant linear trend of self-control strategies and the significant quadratic effect also remained the same substantively. The only interaction that was significant was between self-controlling coping and perceived control. This moderation is such that for those who experienced more control, greater use of self-controlling coping strategies resulted in worse physical functioning; however, for those who experienced lower levels of control, higher levels of self-control predicted better physical functioning (Figure 1).

**Figure 2.**
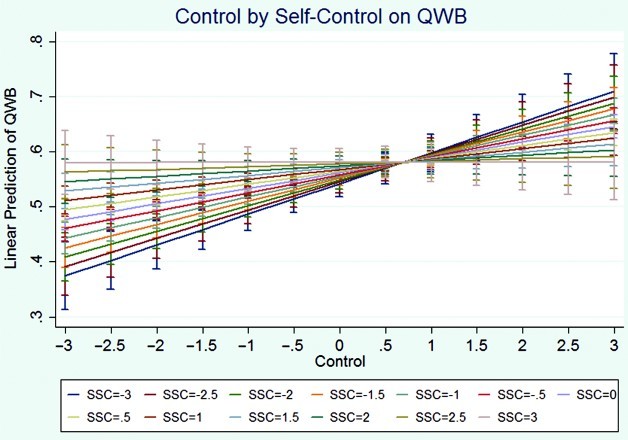
Visual representation of the interaction effect between self-control and control on QWB. Note: QWB,  quality of well-being; SSC, standardized self-control.

Finally, in the third-level model for QWB, it was predicted by employment, age, seeking social support, accepting responsibility, problem-solving, distancing, self-control, and control, *F*(11, 463) = 19.77, *p* < .001, *R* ^2^ = 0.3079, RMSE = 6.4211. For this model *only*, the Breusch–Pagan test for heteroscedasticity showed that the errors were not identically distributed, *χ* ^2^(1) = 10.53, *p* = .001. Thus, a regression analysis using robust standard errors was performed. Controlling for the other variables in the model, those who were employed, who were older, who sought social support less, who accepted responsibility less, who distanced more, and who felt more control experienced higher QWB. The linear relationships between problem-solving and self-control remained non-significant. However, both of these were related to QWB significantly in quadratic form: problem-solving and self-control, and they remained substantively the same as they were in the second-level model. Again, only one interaction was significant, self-control by control. This moderating effect was identical in pattern to that observed in the physical function model; specifically, for those high in perceived control over their FMS, less use of self-controlling coping was associated with greater QWB; however, for those low in control, more self-controlling coping was associated with greater QWB (Figure 2). See Table 3 for specific tests and statistics for third-level models.

**Table 3.** Level 3 models: Demographic, main effects, quadratic effects, and interaction effects.

	FIQ				QWB			
	*β*	*p*	95% CI	*sr*^2^	*β*	*p*	95% CI	*sr*^2^
Income	−0.0467	<0.001	−0.0722, −0.0212	0.0182	–	–	–	–
Employment	−0.2622	<0.001	−0.3799, −0.1445	0.0269	4.9666	<0.001	3.6702, 6.2631	0.0847
Age	−0.0079	0.004	−0.0133, −0.0026	0.0119	0.0611	0.042	0.0022, 0.1200	0.0064
Distancing	−0.2310	<0.001	−0.2992, −0.1627	0.0620	1.1583	0.002	0.4404, 1.8761	0.0169
Self-Control	−0.0003	0.991	–	–	0.6472	0.117	–	–
Escape-avoidance	0.0844	0.011	0.0196, 0.1491	0.0092	–	–	–	–
Control	−0.2470	<0.001	−0.1902, −0.3037	0.1025	2.8040	<0.001	3.4520, 2.1559	0.1174
Problem-solving	–	–	–	–	−0.2117	0.624	–	–
Seeking social support	–	–	–	–	−0.9776	0.005	−1.6591, −0.2960	0.0118
Accepting responsibility	–	–	–	–	−0.8694	0.014	−1.5631, −0.1756	0.0085
Distancing (quadratic)	0.0634	0.011	0.0146, 0.1123	0.0091	–	–	–	–
Self-control (quadratic)	0.0554	0.003	0.0187, 0.0920	0.0123	−0.7598	0.001	−1.2205, −0.2992	0.0165
Problem-solving (quadratic)	–	–	–	–	0.7317	0.022	0.1059, 1.3574	0.0113
Self-control × control	0.0678	0.013	0.1215, 0.0141	0.0086	−0.9264	0.004	−0.3022, −1.5506	0.0137

Note: QWB, quality of well-being scale; FIQ, Fibromyalgia Impact Questionnaire physical functioning subscale; *sr* ^2,^  squared semi-partial correlation.

## Discussion

Individuals with FMS experience greater difficulties in coping with their condition than do other chronic pain populations (e.g. osteoarthritis [OA]; Zautra, Hamilton, & Burke, 1999), and experience lower coping self-efficacy than do individuals in the general population (Johnson, Zautra, & Davis, 2006). In order to better understand how coping and perceived control contribute to physical functioning and well-being within this population, the present study examined the applicability of the goodness-of-fit hypothesis. Consistent with this hypothesis, participants who reported having lower levels of perceived control over their FMS and also engaged in more self-controlling coping (emotion-focused strategy) experienced greater QWB and physical functioning. The goodness-of-fit hypothesis suggests that using an emotion-focused strategy is most effective when one's level of control over his or her stressor is low, which is largely descriptive of one's experience with FMS symptoms. The WOC scale defines self-controlling coping as explicit efforts to regulate emotions and associated behaviors (Folkman & Lazarus, 1988). This strategy is likely to be most effective when people with FMS perceive chronic symptoms as largely outside of their control. Although self-regulation of emotions and actions may help in coping with a chronic condition like FMS, individuals with FMS exhibit self-regulatory difficulties (Solberg Nes, Carlson, Crofford, de Leeuw, & Segerstrom, 2010) and face persistent challenges with regulating positive emotions, especially during times of stress (Zautra et al., 2005). Given these challenges and the possible benefits of self-controlling coping, individuals with FMS might benefit from self-regulatory training, with an additional focus on increasing the capacity to experience positive emotions during times of illness-related stress. No other interactions were significant, suggesting that the entirety of the goodness of fit hypothesis was not broadly supported within the present study.

Additional findings within the present study provide insight into living with FMS. For instance, higher perceived control, on its own, was related to greater QWB and physical functioning, which is consistent with past research (Stanton et al., 2000; Stiegelis et al., 2003). Perceived coping effectiveness is associated with greater adaptation to chronic pain (Zautra et al., 1999). Living with FMS means living with a chronic condition that has no cure and lacks effective treatment. Individuals with FMS must live with the unpredictability of this condition and its symptoms. The findings of this present study suggest that individuals who perceive having greater behavioral control over FMS symptoms, whether or not this is actually experienced, also experience greater quality of life and functioning. More research is needed to better understand these individual differences and how to increase perceived control over the FMS experience. There is growing research to show that behavioral intervention might positively impact one's symptom experience and therefore perceived control, including mindfulness practice (Grossman, Tiefenthaler-Gilmer, Raysz, & Kesper, 2007) and exercise (Busch, Barber, Overend, Peloso, & Schachter, 2007).

Several additional coping strategies were found to affect physical functioning and quality of life within this sample. First, physical functioning was higher among those who reported using less escape-avoidance coping. *Escape-avoidance* (emotion-focused) coping is defined as efforts to behaviorally or emotionally avoid a perceived stressor. Individuals with FMS are more likely to use avoidant coping strategies than are those with OA, another chronic pain population (Zautra et al., 1999). It has been suggested that greater use of avoidance strategies might be explained by the qualitatively different experiences of pain among the two groups (i.e. FMS pain is largely unpredictable, with no physical cause) and the presence of chronic fatigue among those with FMS (Zautra et al., 1999). Individuals with FMS may continue to use escape-avoidance coping strategies over time, despite its low overall effectiveness, because this strategy may provide a temporary, in-the-moment relief from the negative feelings evoked by a stressor (e.g. FMS symptoms); however, this form of coping is concurrently more likely to lead to intrusive emotional experiences and heightened distress (Middendorp et al., 2008; Zautra et al., 1999). This might be akin to the case of an individual with social anxiety who avoids a social situation. By avoiding, the individual lessens his/her anxiety at that moment; however, his/her social anxiety persists and the individual is no closer to functioning better in social situations. Similarly, individuals with FMS may be positively reinforced to avoid engaging in activities that produce heightened pain and fatigue because they receive some symptom relief in the moment; however, this form of avoidance is likely to be linked to worse physical functioning. More research is needed to better understand the motivation behind the use of this coping technique, despite its negative impact on functioning. Therapeutic efforts directed toward alternative coping strategies might be effective in improving long-term QWB and physical functioning among individuals with FMS (Zautra et al., 1999).

Within the present study, both physical functioning and QWB were higher among those who reported greater use of *distancing* (emotion-focused strategy) to cope. This coping strategy incorporates the use of humor, finding a silver lining, having willingness to experience “fate”, and detachment from the significance of the stressor (Folkman & Lazarus, 1988; Folkman, Lazarus, Dunkel-Schetter, DeLongis, & Gruen, 1986). Distancing has been shown to be an adaptive coping strategy, particularly in situations perceived as negative and unchangeable (Folkman et al., 1986). Focusing attention on modulating one's emotional state, such as in the use of distancing, might be the most effective strategy in coping with an unpredictable condition like FMS. It should be noted that physical functioning was lower among those who reported the highest use of distancing coping. While distancing might be an effective means of coping with a stressor, the more that one relies on such a strategy, the less effective it might become. In fact, the use of this strategy in its most extreme form may be more akin to actively trying to downplay the importance of one's condition and ignore its impact. For instance, items on this scale include “I went on as if nothing had happened”, “I didn't let it get to me”, and “I refused to talk about it”. It is possible that some individual distancing strategies may be more or less effective than others. For instance, “I didn't let it get to me” qualitatively differs from “I refused to talk about it”. More in-depth investigation is needed to better understand the range of, and limitations to, the benefits received from various forms of distancing coping.

Another finding within the present study demonstrated that individuals with FMS who sought less social support experienced greater QWB. Seeking social support was assessed as the strategy of eliciting emotional, informational, and physical support from others. A study conducted by Zautra et al. (1999) showed that individuals with FMS reported experiencing fewer positive social interactions than did individuals with OA. There are a number of possible reasons that some people may choose not to seek social support as a way of coping with FMS. For instance, they might not seek social support as a way to cope with FMS because they do not perceive a need for it. This might be related to the lack of availability, or perceived lack of utility, of informational and practical supports for FMS. Alternatively, people might not seek social support because they perceive their social network as being ineffective for helping them cope with FMS. Chronic pain affects not only the individual, but also the person's support network (e.g. family, friends). Given the unpredictable nature of FMS and the difficulties individuals experience in physical functioning, their support network might be less emotionally and/or practically supportive over time as the effects of the person's condition take a toll on the relationship. In a previous study, we found that the quality of social support received was an important predictor of psychological and physical well-being among people with FMS (Franks, Cronan, & Oliver, 2004). Specifically, when people with FMS report receiving high-quality social support, they are likely to experience less mood disturbance and depression, as well as greater self-efficacy for coping with FMS and greater well-being (Franks et al., 2004). Other evidence suggests that interventions designed to enhance the quality of social support are related to improved self-efficacy and reduced feelings of helplessness (Oliver et al., 2001).

In summary, the findings from this study provide important insights into the FMS experience. Although the goodness-of-fit hypothesis was not supported across all coping styles, the one significant interaction between self-controlling coping and control is consistent with this theory of stress and coping. This finding suggests that engaging in emotion-regulation strategies might be beneficial for people with FMS who perceive having limited control over their illness experience. In addition, interesting individual effects emerged, such as the benefits of distancing coping and having perceived control over FMS for QWB and physical functioning. Ultimately, the information collected within the present study is cross-sectional. Participants were asked about coping and control in general rather than specific to certain situations. This procedure poses challenges for interpretation, and the results likely reflect a multidirectional relationship among all the factors. Coping with chronic pain is complex, and the coping strategies might lead to either beneficial or harmful effects on physical functioning, depending on the circumstances in which they are employed (Van Damme, Crombez, & Eccleston, 2008). For instance, research suggests that coping with pain through attempted control or reduction of the pain experience might improve functioning, but likely depends on one's actual level of control over that experience (Van Damme et al., 2008). Individuals may employ different coping strategies in different situations, and the success of their behaviors is likely related to their ability to choose different effective strategies as needed, as well as to the relationship between their perceived level of control and their actual level of control across situations (Van Damme et al., 2008).

This study was directed at examining general patterns; therefore, little information was provided regarding the situation-to-situation coping, frequency of coping strategies used, and effects of each strategy on physical functioning and QWB in each situation. To better understand coping within this heterogeneous population, a longitudinal study would be most applicable for assessing coping strategies across individuals, situations, and times. Individuals probably cope more effectively with some FMS symptoms than with others, and the effectiveness of a given type of coping strategy probably changes as a function of time and experience with the illness. It is important for future researchers to produce a clearer picture of the coping strategies that are most frequently used, the situations in which they are employed, and their effectiveness in improving QWB and physical functioning. This information might be particularly important for tailoring psychosocial interventions to help improve functioning and quality of life among individuals with FMS.
